# Neural Coding of Cooperative vs. Affective Human Interactions: 150 ms to Code the Action's Purpose

**DOI:** 10.1371/journal.pone.0022026

**Published:** 2011-07-07

**Authors:** Alice Mado Proverbio, Federica Riva, Laura Paganelli, Stefano F. Cappa, Nicola Canessa, Daniela Perani, Alberto Zani

**Affiliations:** 1 Department of Psychology, University of Milano-Bicocca, Milan, Italy; 2 Institute of Molecular Bioimaging and Physiology, CNR, Milano-Segrate, Italy; 3 Vita-Salute University and Division of Neuroscience, San Raffaele Scientific Insitute, Milan, Italy; University of Regensburg, Germany

## Abstract

The timing and neural processing of the understanding of social interactions was investigated by presenting scenes in which 2 people performed cooperative or affective actions. While the role of the human mirror neuron system (MNS) in understanding actions and intentions is widely accepted, little is known about the time course within which these aspects of visual information are automatically extracted. Event-Related Potentials were recorded in 35 university students perceiving 260 pictures of cooperative (e.g., 2 people dragging a box) or affective (e.g., 2 people smiling and holding hands) interactions. The action's goal was automatically discriminated at about 150–170 ms, as reflected by occipito/temporal N170 response. The swLORETA inverse solution revealed the strongest sources in the right posterior cingulate cortex (CC) for affective actions and in the right pSTS for cooperative actions. It was found a right hemispheric asymmetry that involved the fusiform gyrus (BA37), the posterior CC, and the medial frontal gyrus (BA10/11) for the processing of affective interactions, particularly in the 155–175 ms time window. In a later time window (200–250 ms) the processing of cooperative interactions activated the left post-central gyrus (BA3), the left parahippocampal gyrus, the left superior frontal gyrus (BA10), as well as the right premotor cortex (BA6). Women showed a greater response discriminative of the action's goal compared to men at P300 and anterior negativity level (220–500 ms). These findings might be related to a greater responsiveness of the female vs. male MNS. In addition, the discriminative effect was bilateral in women and was smaller and left-sided in men. Evidence was provided that perceptually similar social interactions are discriminated on the basis of the agents' intentions quite early in neural processing, differentially activating regions devoted to face/body/action coding, the limbic system and the MNS.

## Introduction

Understanding another person's behavior requires the ability to automatically understand actions and intentions on the mere basis of bodily language. Action processing must also be fast, in order to provide quick reactions to potentially aversive agents, such as recognizing a threat from another person (“is this man trying to hit me?”). An increasingly large amount of neuroimaging data point to the human mirror neuron system (MNS), including the inferior frontal gyrus (IFG), the inferior parietal lobule (IPL), and the posterior part of superior temporal sulcus (pSTS), as the primary neural circuit engaged in action intention understanding [1]–[3]. The principle is that viewing another person's actions activates sensory-motor neurons in the MNS, which is assumed to provide a link between action execution and observation, thus also enabling intention understanding. EEG data have consistently demonstrated a mirror activity in the somatosensory cortex, in terms of a mu rhythm desynchronization or suppression, during the recognition of point-light biological motion [4] as well as reaching and grasping hand movements [5].

Yet, data concerning the relationship between the time course of brain activation and the understanding of the intentions of others based on their behavior are scarce and fragmentary. For example, MEG studies have shown that manipulative hand actions and their observation modulate the somatosensory cortex (SI and SII) with an overall latency of 35-ms for SI responses and of 80–90 ms for the SII response, with no difference between manipulating and observing [6]. Similarly, it has been shown that viewing another person's articulatory gestures (mouth movements) activates the left SI cortex by as early as 55 ms [7]. As for the very early effect, it must be considered that these actions are quite basic and not elaborated, and the somatosensory cortex merely recognizes the biologically relevant gestures “resonating” in the person's view. Studies on more complex learned or symbolic behaviors hint to a much later stage for the processing of the action's purpose. For example, event-related potential (ERP) data have shown that meaningless vs. meaningful hand postures (e.g., the sign for “victory,” or the sign for “OK”) are discriminated at about 400 ms post-stimulus, as indexed by an increase in the right anterior frontal N400 response to meaningless gestures [8]. Similarly, Shibata and co-workers [9] recorded ERPs to appropriate or inappropriate passive/received hand actions. They found a parietal N400 (later spreading at the anterior sites) that was greater in response to inappropriate gestures. Again, Bach et al. [10] investigated ERPs during the evaluation of the appropriateness of tool use actions performed by one person and found that spatially inappropriate tool use actions (e.g., the presentation of a picture showing a hand holding a coin vertically after the presentation of a picture showing a slot for a coin that was horizontal) elicited left lateralized N400s. Although these studies used stimuli depicting actions more complex than the ones used for MEG recording, they only involved a single body part (hand or arm), and the pictures lacked the representation of the whole body of the agent, their social or environmental context, as well as any affective information (i.e., body language and facial expressions). Similarly, in a recent electrophysiological study [11], VEPs were recorded to visual frames showing either an object or a hand interacting with it while viewers were verbally asked to try to understand the intention of the agent. The results showed a strong activation of the left IPL between 200–220 ms, which was interpreted as the stage of intention understanding. This result was also discussed in the context of the role of the IPL, which is often damaged in apraxia patients, in action representation.

Aiming to study the neural circuits underpinning the comprehension of complex human behavior, we recently performed an ERP study [12] in which we compared the perception of congruent and recognizable behavior (e.g., a young woman trying shoes on in a shop) with an incongruent action lacking a comprehensible goal (e.g., a businesswoman balancing on one foot in the desert). The data provided evidence of an early coding of the action's purpose (∼250 ms), especially in females, who also exhibited larger responses. The data also provided evidence of the specific involvement of the IPL, left IFG, left and right premotor areas, right cingulate cortex, right STG and extra-striate cortex according to swLORETA inverse solutions. These data are consistent with those from similar studies in the literature [13].

In the present study, rather than using meaningless actions, we sought to investigate the neural processing of two types of actions characterized by a clearly distinguishable, but radically different goal: pursuing a common goal requiring cooperation between conspecifics (such as lifting a heavy item), or establishing emotional contact without a further goal (not necessarily involving physical contact), which is an essential behavior for social animals.

Few studies have specifically investigated the neural basis of action comprehension during the perception of human scenes in which 2 agents were engaged in a cooperative or affective interaction. Hider and Simmel [14] were the first to show short clips in which geometrical entities (2 triangles and a circle) moved outside and inside a rectangle. These investigators found that children were inclined to describe the figure movements in terms of the cooperative or affective intentions of the agents. More recently, various imaging studies recorded brain activity during the perception of similar configurations: geometrical items displaying social or affective interactions [15]–[17]. Functional magnetic resonance imaging (fMRI) and positron emission tomography (PET) studies point to several brain regions that are active during visual tasks that make use of Heider-and-Simmel animations. These areas include, among others, the posterior part of the right superior temporal sulcus, the parieto–temporal junction, the fusiform face area, and the medial prefrontal cortex. In other studies, fMRI scanning was performed while participants played a cooperation game with a human agent (e.g., cooperate with the experimenter to shape the two sticks of the box in either an angle or a straight line)[18], played two-person “trust and reciprocity” games with both human and computer counterparts for cash rewards [19], or played the “Prisoner's Dilemma” Game with another person [20]. In other studies, social interactions were represented by means of schematic agents depicted by point-lights [21], which were interacting with each other (showing something on the ground) or moving by themselves (jumping, raising a leg). The first type of interaction was named “social” and the second type was named “non-social.” fMRI recording showed a stronger activation of the left temporo/parietal junction, the right anterior superior temporal sulcus (STS) and the dorsal part of the medial prefrontal cortex (MFPC) when viewing the social vs. the non-social interactions.

Although insightful, these studies are based on non-realistic agents that are quite schematic and barely resemble real individuals. These studies also do not provide visual stimulation to neural structures devoted to processing the human figure (body and face), such as the face fusiform area [22] and the extra-striate body area, which are also responsive to action processing, including the action's goal ( [23] and [24]).

The aim of the present study was therefore to investigate the time course and the cerebral mechanisms involved in the neural coding of ecologic and realistic human scenes depicting cooperative interactions (in which two persons are pursuing a common goal), as opposed to perceptually similar interactions where the only goal is to enter into affective contact with each other (affective interaction). Both behaviors are typical of the human repertoire, are spontaneously performed both by adults and young individuals of both sexes, and are universally recognizable on the basis of silent body language. Importantly, actions showing a complex human behavior were presented rather than simple reaching/grasping/hitting arm-based movements [11] or geometrical agents [25]. We aimed to establish how early during neural processing the action's goal is coded.

Since the 2 types of affective vs. cooperative interactions only differed for the diverse agents' intentions (and not for perceptual characteristics) we assumed that they will be associated with a substantially similar ERP morphology (i.e., series of positive and negative peaks) except for those components reflecting the activity of neural structures subserving intention understanding. In the same line of thought, the temporal latency corresponding to the first significant difference in the amplitude of the bioelectric responses to the 2 types of actions would correspond to the processing time required to discriminate the action's purpose. Other studies have shown, for example, that the parietal N2 response (150–280 ms), whose neural generators includes regions of the so-called “human mirror-neuron system (MNS)” (inferior/parietal, left inferior/frontal, left and right premotor areas, right cingulate cortex, right superior/temporal and extra-striate cortex) is strongly modulated by the action's purpose. In the present study, we wished to determine whether an earlier ERP response, namely the occipito/temporal N170, known to reflect the processing of configurational [26], [27], affective [28] and even social [29] face and body properties was affected by stimulus content. Indeed, there are compelling evidence that extra-striate area is involved in the action processing, including the action's goal ( [23] and [24]).

We also determined whether there are sex differences in the time course and neuroanatomical substrates of functional circuits involved because previous studies have suggested a gender difference in the processing of social interactions [12], [25], [30]–[32]. More specifically, a sex difference has been shown in the ability to understand the others' intentions [12] or to comprehend the others' emotional state. This difference has also been related to a neuro-anatomical dimorphism, with females having a significantly larger gray matter volume in the pars opercularis and inferior parietal lobule than males, and therefore a possibly more responding mirror neuron system [33]. In this line of research, Cheng and coworkers [34] measured the electroencephalographic mu rhythm at central sites (C3, Cz, and C4) as a reliable indicator of human mirror-neuron system activity when female and male participants watched either hand actions or a moving dot. The results showed significantly stronger mu suppression in females than males when watching hand actions compared to moving dots. Because mu rhythm results from the spontaneous firing of the sensorimotor neurons in synchrony when individuals execute an action or observe an action performed by another individual, the authors interpreted their data in terms of a gender difference in the mirror activity during action observation. The hypothesis of a sex difference in MNS responsivity was therefore tested (although not being it one of the primary research goal of this study) by comparing the brain's ability to automatically discriminate perceptually similar scenes on the basis of the agents' intentions.

## Methods

### Participants

Thirty-five university students (17 males and 18 females) ranging in age from 20 to 35 years (mean age  = 21.81 years, SD = 2.1) volunteered in this experiment. All participants had a normal or corrected-to-normal vision with right eye dominance. They were strictly right-handed as assessed by the Edinburgh Inventory, and none of them had any left-handed relatives. Experiments were conducted with the understanding and written consent of each participant according to the Declaration of Helsinki (BMJ 1991; 302: 1194), with approval from the Ethical Committee of the Italian National Research Council (CNR) and in compliance with APA ethical standards for the treatment of human volunteers (1992, American Psychological Association). Subjects gained academic credits for their participation. Data from 4 men and 4 women were subsequently discarded because of excessive eye-movements or EEG artifacts. The ovarian cycle of female participants was ascertained and matched across subjects (see Table 1). The 60-item Empathy Quotient (EQ) [35] was administered to assess empathic capacity in men and women. No significant sex differences were found (Men = 52, women  = 51.7).

**Table 1:** Matching of female participant characteristics related to their ovarian cycle.

Hormonal contraceptive	Yes	No	Total
# Ss	8	7	15
Ovarian phase	Follicular(1^st^–14^th^ day)	Luteal(15^th^–28^th^ day)	
# Ss	7	8	15

Number or female subjects that assumed hormonal contraceptives and that were in their pre-ovulatory or post-ovulatory phase of their menstrual cycle at the time of EEG recording. As visible, women were matched across classes so that it can be excluded that higher levels of either estrogen or progesteron (whose concentration changes in the 2 phases) might modulate neural responses to social stimuli similarly in all female participants.

### Stimuli

**Figure 1:**
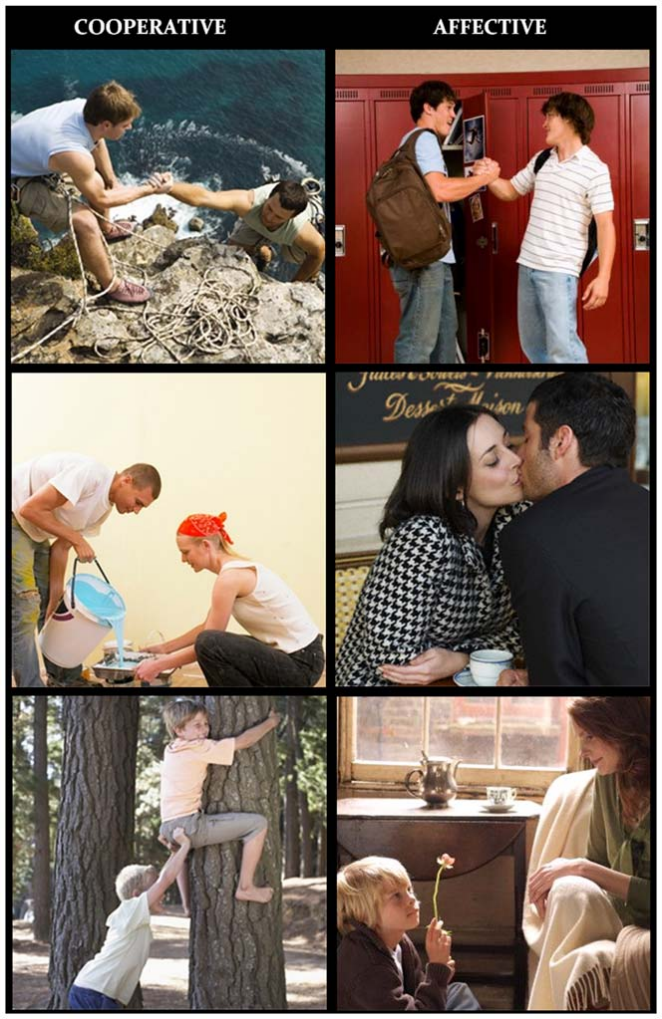
Examples of pictures depicting cooperative vs. affective interactions in young and older agents of both sexes.

The stimulus set was comprised of 260 color pictures depicting males and females of various ages and numbers engaged in goal-directed actions belonging to the typical human repertoire. The pictures were downloaded from Google Images. The action's goal might consist of reaching a common aim (such as lifting a box or dragging heavy furniture), in which case the actions were of the “cooperative” type. In alternative, the goal might be of social nature, to establish an affective contact, or just to relate to someone else (e.g., shaking hands or holding each other), in which case the actions were of the “social” type (see examples in Fig. 1). A total of 130 cooperative and 130 social actions were presented randomly mixed with 44 neutral infrequent targets (landscapes without any visible people). The pictures were 15×15 cm (7° 32′ 33″) in size and their average luminance was 15.48 Foot-lamberts. An ANOVA showed no difference in stimulus luminance as a function of stimulus type. Each slide was presented for 1300 ms at the center of a PC screen with an ISI ranging from 1750 to 1900 ms. The outer background was dark grey.

Stimuli were selected from a wider sample of 310 photos, including 155 items for each category. They were randomly ordered in a PowerPoint file, one per page, and presented to a group of 52 different judges of similar age and educational level as the experimental subjects. Half of the examiners judged the pictures for their cooperative content, while the other half judged them for their social content. The experimenter briefly showed them the pictures (one by one) for a few seconds and asked them to evaluate whether the action presented seemed cooperative (or social) to them by means of a 3-point scale [3 =  very much cooperative (or social); 2 =  vaguely cooperative (or social) 1 =  not at all cooperative (or affective)]. As the judge gave his or her opinion on the photographs, the person administering the test recorded the results for each photograph. The risk of a bias in the responses was minimized by randomly changing the order in which the photographs were presented to each judge. Cooperative pictures were judged as very much cooperative by the 26 judges administering the cooperative survey and not at all social by the 26 judges administering the social survey, and vice versa. Therefore, 50 cooperative and social pictures were discarded because of an insufficient average score (<1.3).

At the end of this process, we were able to select 260 pictures (130 for each category) that were balanced for gender, age, number of persons (see Table 2) and the body part depicted (full-length bodies vs. half-length bodies). In order to have subjects performing a secondary task, 44 further photos depicting common natural or urban landscapes without visible persons (including streets, offices, shops, a public library, the countryside, a seascape, a mountain landscape, etc.) were also included. These pictures were equal to the human pictures in terms of average luminance and size.

**Table 2:** Inter-categorical balancing of sex, number and age of agents depicted in human scenes.

Age	Adults		Children		Both		Total	Total
Scene Content	Aff.	Coop.	Aff.	Coop.	Aff.	Coop.	Aff.	Coop.
Men	8	10	5	6	10	11	23	27
Women	10	10	6	5	15	15	31	30
Both	52	50	6	7	18	16	76	73
Total	70	70	17	18	43	42	130	130

### Task and procedure

The task consisted of responding as accurately and quickly as possible to the presence of landscapes (scenarios without visible persons) by pressing a response key with the index finger of the left or right hand while ignoring all other pictures. The two hands were used alternately during the recording session. The order of the hand and task conditions was counterbalanced across subjects.

Participants were comfortably seated in a darkened, acoustically and electrically shielded test area. They faced a high-resolution VGA computer screen located 114 cm from their eyes. They were instructed to gaze at the center of the screen, where a small circle served as the fixation point, and to avoid any eye or body movements during the recording session. Stimuli were presented at the center of the screen and were randomly mixed in 8 different short runs of 32–36 trials that lasted about 2 minutes each. For each experimental run, the target stimuli varied between 2 and 8 runs. The sequence presentation order differed across the subjects.

### EEG recording and analysis

The EEG was continuously recorded from 128 scalp sites at a sampling rate of 512 Hz by means of an ANT-EEprobe 3.1. system. Horizontal and vertical eye movements were also recorded. Linked ears served as the reference lead. The EEG and electro-oculogram (EOG) were amplified with a half-amplitude band pass of 0.016–100 Hz. Electrode impedance was kept below 5 kΩ. EEG epochs were synchronized with the onset of stimuli presentation. Computerized artifact rejection was performed before averaging to discard epochs in which eye movements, blinks, excessive muscle potentials or amplifier blocking occurred. The artifact rejection criterion was peak-to-peak amplitude exceeding 50 µV, and the rejection rate was ∼5%. ERPs were averaged off-line from −100 ms before to 1000 ms after stimulus onset. ERP components were identified and measured, with reference to the average baseline voltage over the interval from −100 ms to 0 ms, at sites and latency where they reached their maximum amplitude.

The mean amplitude of the occipito/temporal N170 was measured at the PO9, PO10, PPO10h, and PPO9h sites during the 150–190 ms time window. The parietal N2 response was measured at the Pz, P3, and P4 sites during the 160–280 ms time window. Posterior P300 was measured at the same sites (PO9, PO10, PPO10h, and PPO9h) between 250–350 ms post-stimulus. Anterior negativity (N2/N3 deflections) was quantified at the F1, F2, F5, F6, C1, and C2 electrode sites in the 220–500 ms post-stimulus time window.

ERP data were subjected to a multifactorial repeated-measures ANOVA with one factor between (sex: males, females) and 3 factors within groups. The within factors were as follows: scene content (cooperative, affective), electrode (dependent on the ERP component of interest) and hemisphere (left, right) for the ERP data. Multiple comparisons of means were performed by the post-hoc Tukey tests.

*Low Resolution Electromagnetic Tomography* (LORETA) [36] was performed on ERP difference waves at various time latencies. LORETA, which is a discrete linear solution to the inverse EEG problem, corresponds to the 3D distribution of neuronal electric activity that has maximum similarity (i.e., maximum synchronization) in terms of orientation and strength between neighboring neuronal populations (represented by adjacent voxels). In this study, an improved version of the weighted low-resolution brain electromagnetic tomography (sLORETA) was used, which incorporates a singular value decomposition-based lead field weighting: swLORETA [37]. The source space properties were as follows: grid spacing (the distance between two calculation points)  = 5 points; estimated signal to noise ratio (SNR, which defines the regularization; and a higher value for SNR means less regularization and less blurred results)  = 3 points. LORETA was performed on group data and it identified statistically significant electromagnetic dipoles (p<0.05). The larger the magnitude, the more significant the difference in activation between the two compared conditions was.

A realistic boundary element model (BEM) was derived from a T1 weighted 3D MRI data set by segmentation of the brain tissue. The BEM model consisted of one homogenic compartment made up of 3446 vertices and 6888 triangles. The head model was used for intra-cranial localization of surface potentials. Segmentation and head model generation were performed using the ASA (A.N.T. Software B.V., Enschede, The Netherlands) package [38].

## Results

### Occipito/temporal N170 (150–190 ms)

**Figure 2:**
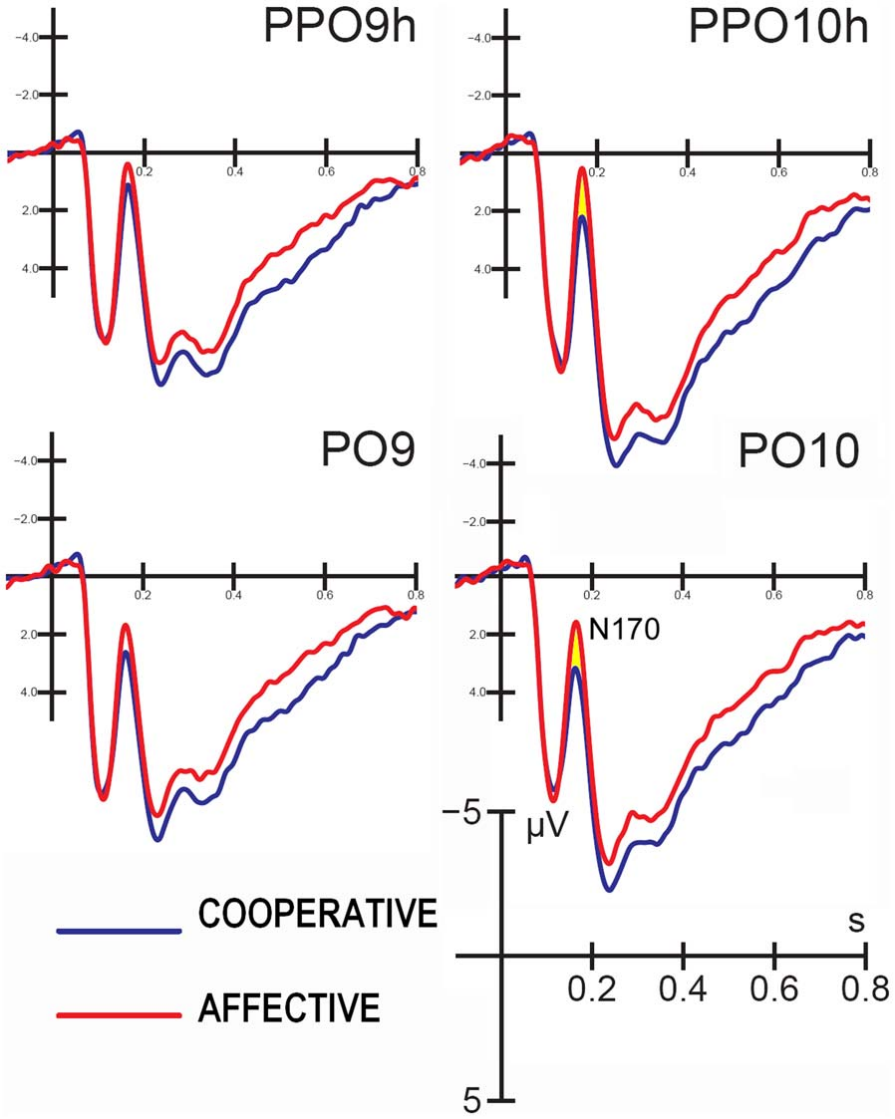
Grand-average ERP waveforms recorded at the left and right occipito/temporal sites in response to affective vs. cooperative actions, independent of the viewer's sex.

The ANOVA performed on the N170 mean amplitude values yielded the significance of scene content (F(1,25)  = 36.41; p<0.000003; ε = 1), which showed greater N170 amplitudes to affective scenes compared to cooperative scenes (AFF. = 1.45 µV, COOP. = 2.55 µV). This result is displayed in Fig. 2. The N170 was greater at the occipito/temporal site than the lateral occipital electrode sites (F(1,25) = 29.29; p<0.00001; ε = 1), as indicated by post-hoc comparisons (PPO9/10h 1.42 µV vs. PO9/10 e PO09 2.58 µV). The further interaction of scene content x hemisphere (F(1,25)  = 23.49; p<0.00005; ε = 1) and relative post-hoc comparisons among means demonstrate that the N170 was larger over the left hemisphere in response to cooperative scenes (LH = 2.24, SE = 0.72; RH = 2.87, SE = 0.6; diff = p<0.0017), while it was bilateral in response to social scenes (LH = 1.44, SE = 0.76; RH = 1.46, SE = 0.61).

**Figure 3:**
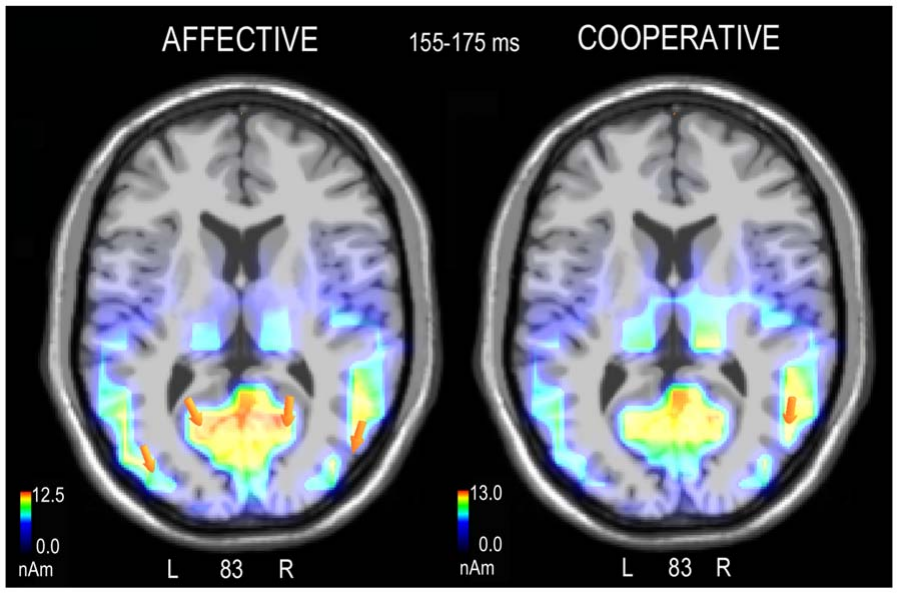
Axial view of N170 active sources for the processing of affective (left) and cooperative (right) human interactions according to the swLORETA analysis during the 155–175 ms time window.

To investigate the effect of scene content on the visual processing of human interactions, two swLORETA inverse solutions [36] were performed on the negative voltage related to affective vs. cooperative processing during the N170 time window (155–175 ms). LORETA analysis (see Table 3 for a list of significant electromagnetic dipoles) showed that the processing of affective gestures was associated with significant activity in the posterior cingulate cortex of the right hemisphere (BA30) and in the right (BA37) and left (BA19) medial occipital gyrus, as visible in the axial section of Fig. 3. On the other hand, LORETA analysis performed on brain activity elicited by cooperative actions was associated with the activation of the right middle temporal/posterior STG, the right parahippocampal gyrus and the right medial frontal gyrus.

**Table 3:** Talairach coordinates corresponding to the intracranial generators, which explain the surface voltage related to the processing of affective and cooperative actions during the 155–175 ms time window.

Magnitude	T-x [mm]	T-y [mm]	T-z [mm]	Hem.	Lobe	Area	BA
AFFECTIVE							
27.8	21.2	−57.9	5.6	R	Limbic	Posterior Cingulate	30
25.5	50.8	−68	4.7	R	O	Medial occipital gyrus	37
23.9	−38.5	−78.2	3.8	L	O	Medial occipital gyrus	19
5.38	1.5	48.2	−17.2	R	F	Medial frontal gyrus	11
2.32	1.5	64.4	16.8	R	F	Medial frontal gyrus	10

COOPERATIVE							
12.90	50.8	−57.9	5.6	R	T	Middle temporal Gyrus, pSTG	21/37
13.01	21.2	−46.8	−2.1	R	Limbic	Parahippocampal Gyrus	19/20
2.85	1.5	64.4	16.8	R	F	Medial Frontal Gyrus	10

According to the swLORETA (ASA) analysis [37]; grid spacing  = 5 mm; estimated SNR  = 3.

### Parietal N2 (160–280 ms)

**Figure 4:**
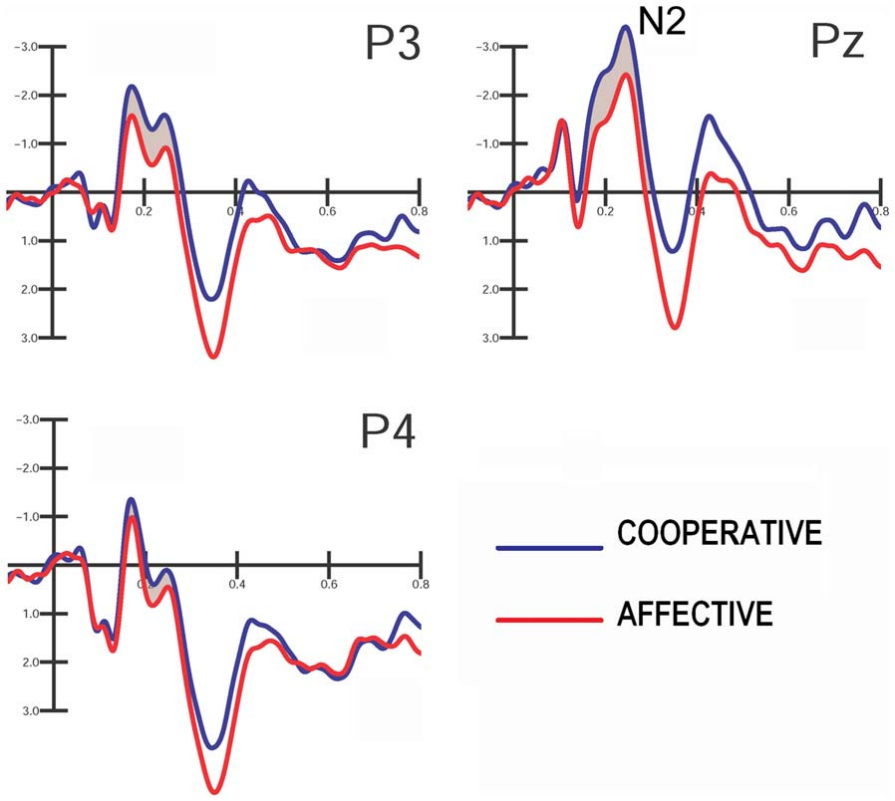
Grand-average ERP waveforms recorded at the left, mesial and right parietal sites in response to affective and cooperative actions, independent of the viewer's sex.

N2 reached its maximum amplitude at parietal sites (Pz, P3, and P4) between 160–280 ms. Statistical analysis shows the significance of scene content (F(1,25)  = 5.04; p<0.03; ε = 1), with greater N2 amplitudes in response to cooperative actions vs. affective actions (COOP. = −1.18 µV; AFF. = −0.75 µV). The electrode factor (F(1.68, 42)  = 22.24; p<0.00001, ε = 0.84) showed that N2 was larger at the midline site (Pz), but with a strong left hemispheric asymmetry (PZ = −1.87; P3 = −1.11 µV; P4 = 0.08 µV), as demonstrated by significant post-hoc comparisons. The further interaction of scene content with the electrode (F(1.4, 35)  = 18.23; p<0.000018; ε = 0.70) showed a non-significant difference in the N2 response between scene types over the right hemisphere, and a significantly larger N2 (p = 0.00014) in response to cooperative (−1.34 µV) vs. affective interactions (−0.88 µV) over the left parietal site. This result is clearly visible in Fig. 4.

**Figure 5:**
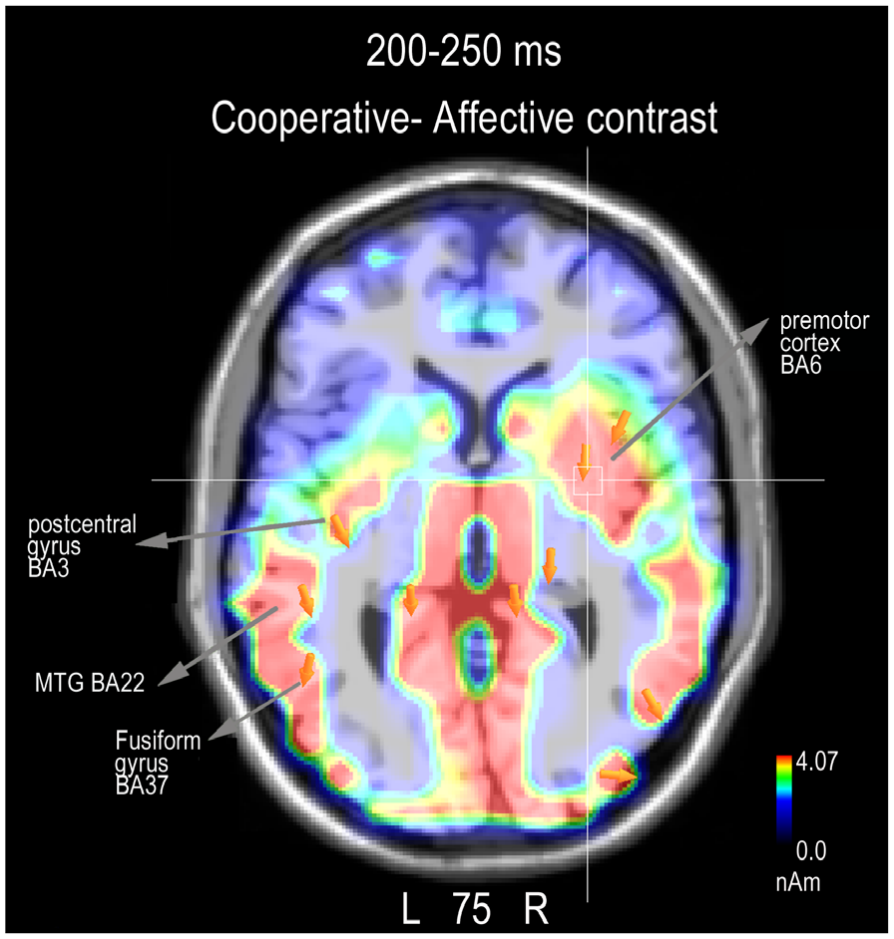
Axial view of N2 active sources for the processing of cooperative minus affective human interactions according to the swLORETA analysis during the 200–250 ms time window.

To locate the possible neural source of the action content effect, two different swLORETA source reconstructions were performed separately for cooperative and affective actions during the 200–250 ms time window, which corresponds to the peak of the parietal N2. The inverse solution showed that the processing of affective actions was associated with electromagnetic activity in a number of left and right hemispheric regions, which are listed in Table 4. These regions include the right fusiform gyrus (BA 37), the left parahippocampal gyrus (BA37), the left post-central gyrus (BA 3), the left and right premotor area (BA6) and the left orbitofrontal cortex (BA10, 11). On the other hand, the processing of cooperative scenes resulted in the activation of partially similar regions (see Table 4), except for a stronger activation of the left fusiform gyrus (BA3), the left post-central gyrus (BA3), the left parahippocampal gyrus, and the left superior frontal gyrus (BA10). The activation was stronger over the right hemisphere over the premotor cortex (BA6). These differences were confirmed by a further comparison performed by subtracting the brain activity (ERPs) evoked by affective actions from that evoked by cooperative actions, and computing a LORETA inverse solution on the difference wave so obtained. The significant electromagnetic dipoles explaining the difference voltage are marked by an asterisk in Table 4 and are visible in Fig. 5.

**Table 4:** Talairach coordinates corresponding to the intracortical generators, which explain the surface voltage recorded during the 200–250 ms time window in response to affective and cooperative actions.

AFFECTIVE (200–250 ms)							
Magnitude	T-x [mm]	T-y [mm]	T-z [mm]	Hem.	Lobe	Area	BA
8.68	40.9	−55.9	−10.2	R	T	Fusiform gyrus	37
6.58	−28.5	−45.8	−9.5	L	Limbic	Parahippocampal gyrus	7
1.88	−38.5	−21	35.7	L	P	Post-central gyrus	3
1.50	−8.5	57.3	−9	L	F	Superior Frontal gyrus	10
1.46	−85	38.2	−17.9	L	F	Rectus	11
1.03	−38.5	2.4	29.4	L	F	Pre-central gyrus	6
0.99	40.9	2.4	29.4	R	F	Pre-central gyrus	6
0.91	−28.5	56.3	−1.6	L	F	Superior Frontal Gyrus	11

COOPERATIVE (200–250 ms)							
Magnitude	T-x [mm]	T-y [mm]	T-z [mm]	Hem.	Lobe	Area	BA
9.62*	40.9	−55.9	−10.2	R	T	Fusiform gyrus	37
7.33*	−28.5	−45.8	−9.5	L	Limbic	Parahippocampal gyrus	37
7.23*	−48.5	−55.9	−10.2	L	T	Fusiform gyrus	37
2.17*	−38.5	−21	35.7	L	P	Postcentral gyrus	3
1.84*	−8.5	57.3	−9	L	F	Superior frontal gyrus	10
1.62	−8.5	38.2	−17.9	L	F	Rectus	11
1.29*	40.9	2.4	29.4	R	F	Premotor cortex	6
1.15	11.3	57.3	−9	R	F	Superior frontal gyrus	10

Power RMS  = 276.2 µV. Asterisks indicate the brain structures that were significantly more active during perception of cooperative than affective interactions, as provided by a LORETA inverse solution (displayed in Fig. 5) applied to the difference-waves obtained by subtracting ERPs to affective from cooperative interactions.

### Posterior P300 component (250–350 ms)

**Figure 6:**
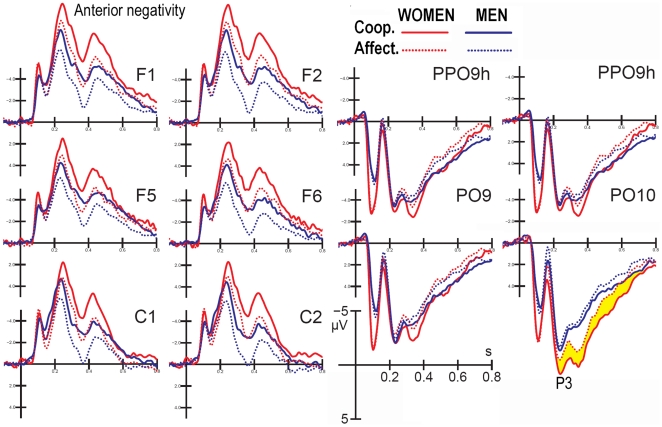
Grand-average ERP waveforms recorded at the left and right dorsal prefrontal, inferior frontal, central, occipito-temporal and lateral occipital sites in response to affective and cooperative actions. The results are analyzed separately for women and men.

This positive deflection was measured at the lateral occipito/temporal sites during the 250–350 ms time window. The ANOVA analysis showed a lateralization effect (F(1,25)  = 11.84; p<0.002; ε = 1), with a larger P300 recorded over the right (RH = 8.75 µV) than the left hemispheric sites (6.72 µV). The P300 was strongly modulated by scene content (F(1,25)  = 18.06; p<0.0002; ε = 1) and was much more positive in response to cooperative actions compared to affective actions in both genders (COOP. = 8.17 µV; AFF. = 7.30 µV). However, a simple affect analysis showed that, while scene content was strongly significant in women (F(1,13)  = 13.07; p<0.003; ε = 1) with a P300 to cooperative actions exceeding 1.21 µV P300 compared to affective actions (Women: COOP. = 8.80, SE = 1.21; AFF. = 7.59 µV, SD = 1.02), the effect was less significant in men (F(1,12)  = 5.56; p<0.03; ε = 1), with a content-related difference of only 0.53 µV (Men. COOP.: 7.54, SD = 0.73; AFF. = 7.01, SD = 0.64). This sex difference is highlighted in Fig. 6.

### Anterior Negativity (220–500 ms)

The anterior negativity was recorded at anterior sites (F1, F2, F5, F6, C1, and C2) during the 220–500 ms time window. The anterior negativity was of greater amplitude at medial frontal sites (medial frontal = −4.93 µV; inferior frontal F5–F6 = −4.3 µV; central C1–C2 = −3.59 µV), as demonstrated by the significance of the electrode (F(1.31, 32.82)  = 16.27; p<0.00001; ε = 0.65). ANOVA analysis showed a significant effect of scene content (F(1,25)  = 62.28; p<0.000001; ε = 1), with a wider anterior negativity in response to cooperative scenes compared to affective scenes (COOP. = −5.06 µV; AFF. = −3.48 µV). The interaction of hemisphere with sex (F (1,25)  = 5.28; p<0.03; ε = 1) and the relative post-hoc comparisons showed a bilateral (and greater) anterior negativity in women (−5.32 µV) and a much smaller and left-sided (p<0.014) negativity in men (−3.15 µV).

## Discussion

In this study, we aimed to investigate the brain correlates of the processing of an action's goal by directly comparing the neural correlates of cooperative vs. affective action processing. To do so, we presented hundreds of realistic scenes depicting 2 persons of different ages and sexes engaged in a behavior belonging to the typical human repertoire in the context of an urban or natural environment. Viewers were male and female university students attentively perceiving these pictures but engaged in a secondary perceptual task. The secondary task (detecting an inanimate landscape) was introduced to avoid a conscious awareness of two types of behavior. Indeed, no subject revealed knowledge about the two-fold nature of the behavior observed at the end of EEG recording; this finding is quite understandable as the two types of interactions did not differ at the perceptual level because people could be spatially very close or far from each other, smiling or neutral, and gesticulating/moving or resting/quiet. Because the cooperative and affective actions were matched for a number of perceptual characteristics, except for the real goal of the human interaction (“are you trying to help me to lift this sofa or are you just entering into contact with me?”), the contrast between neural processing of the two types of actions allowed us to shed some light on the neural mechanisms promoting the comprehension of the other intentions and the exact time course by which this information is automatically extracted from visual inputs and made available for further processing. Thus, time-locked ERP responses were identified and measured over occipito-temporal sites along the ventral stream (N170 and P300 components), over the parietal area (160–280 ms), and at frontal sites (late anterior negativity).

The N170 data provided evidence of an early processing of affective scene content. Indeed, the brain response was of greater amplitude in response to affective stimuli compared to cooperative stimuli between 150–190 ms. This finding agrees with many studies in the literature supporting an early coding of stimulus affective valence for both faces [39], [40] and complex human scenes [41], [42].

Overall, the N170 was of greater amplitude over the right hemisphere and in response to affective pictures. In agreement with the surface ERP data, the swLORETA inverse solution displayed a strong activation of the limbic system and especially the right posterior cingulate cortex in response to affective pictures compared to cooperative pictures. It is known that both anterior and posterior cingulate cortices are involved in emotion processing [43], [44], in the subjective evaluation of events, and in their emotional significance. Specifically, the ventral posterior cingulate cortex is involved in the coding of visual stimulus emotional content [45], thus supporting our finding of a greater activation of the posterior cingulate (BA30) in response to affective vs. cooperative actions. In our study, the swLORETA source reconstruction identified other generators (besides the cingulate cortex), which included the medial occipital gyrus (BA19 and BA37) and the right medial frontal cortex (BA10/11), which are possibly involved in the processing of both faces and bodies, thus explaining the N170 surface voltage. The involvement of prefrontal neurons in the early coding of social information is supported by available literature. In a combined ERP/fMRI study [46] face recognition was associated with haemodynamic increases in fusiform, medial frontal and orbitofrontal cortices. Again, in a very recent MEG study [47] it was found an activation of the right prefrontal cortex that was maximum at 240 ms for inverted faces but was very pronounced also at 170 ms of latency. Quite consistently, face responsive neurons have been identified in the prefrontal cortex of rhesus monkeys [48].

It should be noted that the right hemispheric generator in the visual cortex (Middle Occipital gyrus, MOG) had a stronger magnitude (in nA) compared to the left hemispheric generator. This finding agrees with studies showing a strong hemispheric asymmetry in the face-related [39], [49]–[51] and body-related [52] N170 electromagnetic response. However, such an asymmetry may reflect the numerous presence of male individuals in the experimental sample, since sex differences in the lateralization of face-related visual processing exist, with more bilateral processing in women and right-sided lateralization in men [53], [54].

The early coding of cooperative pictures was instead associated with the activation of the right MTG/pSTG (BA21), which was the strongest generator. The early coding of cooperative pictures was also associated with the right parahippocampal area possibly involved in the processing of scenes and places (PPA), which were more relevant for comprehending the action's goal than for affective interactions, that are more centred to the human body and facial expressions). The early coding of cooperative pictures was also associated with the medial frontal cortex. Both this region [55] and especially the rSTG have been repeatedly described as described as being primarily involved in perceiving biological motion [56], [57] and understanding of others' goals and intentions [58], [59].

The analysis of the time course of brain processing indicates that the coding of scene content and possibly of the action's goal was faster for affective scenes than cooperative scenes. Indeed, the subsequent centro-parietal response (N160–280) was the first component that displayed a larger potential to cooperative actions compared to affective actions, particularly over the left hemisphere. The swLORETA inverse solution provided evidence of a strong parietal involvement of the left post-central gyrus along with the right pre-central gyrus (BA6). The left hemispheric symmetry in IPL activation strongly agrees with the LORETA inverse solution from Ortigue et al. [11], which measured VEPs to hand-objects interactions. According to Grezes and Decety [60], the precentral gyrus is involved in the mental simulation of human actions. The *embodied theory* of action [61] predicts that simulation is based on the activation of the somatosensory cortex. Furthermore, several MEG [6], [7] and fMRI studies [62] suggest that the somatosensory cortex is particularly active during the observation of actions. Some studies [63]–[65], indeed, have explicitly included the somatosensory cortex in the fronto-parietal human mirror system devoted to the comprehension of human actions. As for the precentral gyrus (BA6) activation found in response to cooperative actions, many studies have demonstrated the role of the premotor cortex in action comprehension [66], [67], in the coding of action motor schema [68] and in mental simulation of actions[69]. On the other hand, the activation of more anterior brain regions (left and right superior frontal gyri) may be linked to their role in the automatic comprehension of the action purpose for both affective and cooperative interactions [55], [70].

Going further with the time course of information processing within the 250–350 ms time window, a positive occipito/temporal P300 was of greater amplitude in response to cooperative actions compared to affective actions. A simple effect analysis revealed a greater discriminative effect in women than men. It is interesting to note that a recent fMRI study [71] performed with the same experimental paradigm, but on different subjects, provided evidence of a sex difference in neural activation as a function of the type of action. Cooperation-specific activity engaged mostly limbic and reward-related areas (right ventral striatum and caudal orbitofrontal cortex) in males, while areas associated with bilateral fronto-parietal mirror-activity (EBA, pSTS, rostral portion of the inferior parietal lobule and premotor cortex) were more strongly activated by the same condition in females than males. It is possible to hypothesize that the specific pattern of activation in the female brain reflects, to a greater extent, a resonating system supporting the comprehension of the action's intentions. Indeed, the superior temporal sulcus and the ventral premotor cortex are part of the so-called human mirror neuron system (MNS) [66], [67], [72]. It is likely that the MNS mirrors the actions and experiences of others with one's own actions and experiences, thus providing a key to understanding the intentions of others [70], [73].

The gender difference in the comprehension of the actions' purpose also fits with previous evidence of a greater empathic attitude in females [12], [41], [74]–[76]. Kaplan and Iacoboni [77] suggest that the MNS supports a simulation system devoted to the understanding of the intentions of others and that this system is linked to other social competence functions, such as empathy. In the literature, some neuroscientific evidence of a sex difference in the responsiveness of the MNS to human actions was recently demonstrated. In particular, Cheng and collaborators [33] used a voxel-based morphometry analysis to show that young adult females had significantly larger gray matter volume in the pars opercularis and inferior parietal lobule than matched male participants. The authors interpreted their data as an index of neuroanatomical sex differences in the human MNS. They also suggested that the network of the human mirror-neuron system is strongly linked to empathy competence.

In the present study, Anterior Negativity modulated the amplitude of fronto-central N2 and N400 deflections that were much greater during the processing of cooperative actions. This higher order and later involvement of prefrontal brain regions in the processing of socially-relevant information has been previously reported, for example in the processing of social relations by medial prefrontal cortex in [55]. Again, the roles of the frontal areas (the prefrontal and orbitofrontal cortex) in higher order cognitive functions, such as social reasoning and decision making, have been determined [44]. The LORETA inverse solution performed on ERP data showed that the medial and superior frontal gyri (BA10/11) were indeed active as early as 170 ms post-stimulus during action processing. However there was a difference in their activation as a function of the type of interaction and along the time course of neural processing. At N170 level the medial frontal cortex was activated over the right hemisphere, and more strongly to affective than cooperative interactions, whereas at N2 level the superior frontal gyrus was activated over the left hemisphere, more strongly to cooperative than affective interactions.

The sex difference in hemispheric lateralization relative to the scalp distribution of anterior negativity is also interesting. While the anterior negativity was bilateral in women, it was strongly left-sided in men. This sex difference in lateral preference and hemispheric lateralization is well documented for a variety of stimuli in the literature. For example, in an ERP study on the emotional processing of facial expressions, Proverbio et al. [53] found a smaller degree of lateralization of face-devoted ERP responses (P1 and N170) in women compared to men. Several studies have found a bilateral vs. right-sided bias in structural brain asymmetry (e.g., [78]) and in the emotional coding of visual information [79], [80]. Overall, our results agree with many studies that show differences between men and women in the degree of lateralization of cognitive and affective processes. Substantial data support greater hemispheric lateralization in men than women for linguistic tasks [81] and for spatial tasks [82]. Gender differences have also been found in the lateralization of visual-spatial processes, such as object construction and mental rotation tasks [83], in which males are typically right hemisphere (RH)-dominant while females are bilaterally distributed.

### Conclusions

The present ERP data suggest the existence of a neural circuit that strongly responds to visual scenes depicting human interactions and is capable of discriminating goal-directed cooperative vs. affective actions. In particular, affective scenes were processed earlier than cooperative scenes, as indicated by the latency of early N170 modulation. The LORETA analysis identified a strong focus of activation in the cingulate cortex (which is known to provide the affective connotation to visual coding), the medial occipital cortex and the face fusiform gyrus (possibly devoted to face and body processing) during the perception of affective scenes, and the right medial frontal cortex.

The specific processing of a cooperative purpose did not emerge before 200 ms and progressed until 500 ms post-stimulus, as indexed by the modulation of parietal N200, P300 and anterior negativity, which were of greater amplitude in response to cooperative pictures compared to affective pictures. Cooperative scenes seemed to initially activate the pSTG and the medial frontal cortex, and neural populations belonging to the fronto-parietal neuron-mirror system were activated thereafter ([1], [12], [84]). The LORETA analysis identified the sources of activation for the processing of cooperative actions over the left parietal cortex and the left and right premotor areas (BA6)[71], thus indicating that the mirror neuron system (MNS) is more strongly activated by cooperative, than affective, actions. This result is consistent with the MNS being involved in the visuo-motor transformation of actions and action representation (parietal N2). Later on, the premotor and prefrontal areas are involved in more complex social processing (P300 and Anterior Negativity).

The analysis of posterior P300 responses also suggests a sex difference in the processing of the two scene types. Indeed, a larger inter-category difference was found in women compared to men, suggesting improved comprehension of unattended social scenes. This finding is possibly related to women's supposed increased interest in conspecifics [85].

Finally, our results highlighted a different pattern of hemispheric lateralization as a function of scene content and viewers' gender. The N170 response was greater over the left hemisphere, compared with the right one, only in response to cooperative scenes, while the response was bilateral in response to social scenes. Again, the N2 amplitude showed a lack of scene content coding over the right hemisphere and a significantly larger N2 in response to cooperative vs. socially-aimed interactions over the left parietal site. Consistently, the LORETA inverse solution provided evidence of a stronger activation of left-sided regions during the processing of cooperative actions between 200–250 ms (left fusiform gyrus, BA37, left parietal cortex (BA3), and left para-hippocampal gyrus), along with a stronger activation of the right premotor cortex (BA6). These results are in agreement with previous investigations [11]. Women showed a larger response that was discriminative of action intentions compared to men at the posterior P300 level (250–350 ms) and at the anterior negativity level (220–500 ms). In addition, the discriminative effect was bilateral in women, and much smaller and left-sided in men suggesting that this finding may be related to the supposed greater responsiveness of the female vs. male MNS [12], [25], [33], [34], [74]. One potential limitation of this study, however, is the sample size, which was not so conspicuous for analyzing sex-related differences. As a consequence, it should be at least considered that some null findings might be due to lack of power.
